# Bio-Functional Potential and Biochemical Properties of Propolis Collected from Different Regions of Balochistan Province of Pakistan

**DOI:** 10.1155/2022/7585406

**Published:** 2022-10-05

**Authors:** Ali Akbar, Zareen Gul, Saliha Aziz, Muhammad Bilal Sadiq, Jahangir Khan Achakzai, Shazia Saeed, Su Hlaing Chein, Hassan Sher

**Affiliations:** ^1^Department of Microbiology, University of Balochistan, Quetta Balochistan, Pakistan; ^2^Department of Botany, University of Balochistan, Quetta Balochistan, Pakistan; ^3^School of Life Sciences, Forman Christian College (A Chartered University), Lahore, Pakistan; ^4^Disipline of Biochemistry, Department of Natural and Basic Sciences, University of Turbat Kech, 92600 Balochistan, Pakistan; ^5^Spectrum-Sustainable Development Knowledge, Yangon 11111, Myanmar; ^6^Centre for Plant Sciences and Biodiversity, University of Swat, Charbagh, 19120 Khyber Pakhtunkhwa, Pakistan

## Abstract

Propolis is a well-known resinous natural substance collected by honeybees (*Apis mellifera* L.) from plants exudations. Variations in chemical composition of propolis are due to different sources from which it is collected and change in climate and geographical location. In this study, different propolis samples were collected from different regions of Balochistan and examined for its chemical composition, total phenolics and total flavonoid contents, and antioxidant potential by using DPPH radical scavenging assay and antimicrobial activity. Bioactive components analysis revealed the presence of steroids, carbohydrates, flavonoids, coumarins, cardiac glycosides, quinones, anthraquinones, terpenoids, tannins, and phlobatannins at different levels. The total phenolics contents were ranged from 2.9343 ± 1.247 to 6.0216 ± 2.873 mg GAE g^−1^, and flavonoid contents were found to be 0.1546 ± 0.087 to 0.6586 ± 0.329 mg QE g^-1,^ respectively. The antioxidant ability of each extract was analyzed by their concentration having 50% inhibition (*IC*_50_). The propolis sample P3 possessed lower *IC*_50_27.07 ± 0.73 mg mL^−1^ with higher % inhibition of DPPH radical, and P8 showed lower % inhibition by having *IC*_50_84.43 ± 2.07 mg mL^−1^. The antibacterial activity of all samples was analyzed against a wide group of bacteria including *Escherichia coli*, *Staphylococcus aureus*, *Pseudomonas aeruginosa*, and *Klebsiella pneumonia* and propolis extract (P4) was highly active against *Klebsiella pneumoniae* with the maximum diameter of zone of inhibition 20.33 ± 1.52 mm, and propolis extract (P3) showed maximum zone of inhibition against *Escherichia coli*19.06 ± 1.90, while propolis extract (P2) was found less active with minimum diameter of zone of inhibition 7.46 ± 1.50 mm. The antifungal activity of extract was considered as active against the fungal species. Propolis extract (P3) showed 82% of zone of inhibition against *Aspergillus Niger*, and propolis extract (P1) was highly active against *Aspergillus parasiticus* with 80% of zone of inhibition. By comparing the vibration frequencies in wave numbers of the sample spectrograph acquired from an FTIR spectrophotometer, the functional groups present in the extracts were identified. The presence of seven elements (Fe, Zn, Mn, Ni, Pb, Cd, and Cr) was analyzed through atomic absorption spectrophotometer. The obtained concentrations were within the permissible ranges established by the World Health Organization. The GC-MS analysis revealed the presence of 80 different compounds belonged to different classes. The obtained results confirmed the imperative potential of propolis which can be used in various biological applications.

## 1. Introduction

Honeybees like Apis mellifera L. collect propolis, a sticky adhesive natural substance, from the buds, leaves, and other parts of plants and trees [1]. Propolis is rich in natural compounds which mainly depend on the type of plant accessible to the bees, geographical origin, and collection season. Propolis contains phenolic compounds (flavonoids, phenolic acids, and their esters) that have promising biological actions such as antibacterial, anti-inflammatory, and antioxidant potential [2]. Propolis usually comprises of 50% resin and plant balm, 30% wax, 10% volatile oils, 5% pollen, and 5% other organic residues [3]. More than 300 chemicals have been found in propolis samples from various geographical origins, according to current investigations [4]. Both volatile and nonvolatile fractions of propolis from various botanical and geographic origins were discovered to contain a wide range of chemical compounds, including aldehydes, organic acids, esters, hydrocarbons, cyclic compounds, terpenes, flavonoid aglycones, phenolic acids and their esters, phenolic aldehydes, alcohols, ketones, sesquiterpenes, quinones, and coumarins [5].

The widespread use of propolis in modern and traditional medicine has heightened interest in its chemical composition. Because of the presence of numerous components, propolis is becoming more popular as a natural medication and additional food. Propolis' chemical ingredients defend against oxidative stress-related chronic diseases such as cancer and metabolic disorders [4]. It serves as a body defense agent against free radicals; propolis as a natural substance has promising antioxidant potential [2]. Previous studies reported the antibacterial potential of propolis extract against *Mycobacterium tuberculosis*; moreover, the extract synergized the effect of established anti tubercular drugs such as isoniazid [6]. Propolis and its components were also reported with promising anti-*Helicobacter pylori* activity. Propolis is used in a wide range of cosmetic products such as creams, shampoos, and lotions [7]. Propolis composition may vary with botanical and geographical origins. In this study, propolis collected from different regions of Balochistan province of Pakistan was analyzed for chemical composition and bioactive potential.

## 2. Materials and Methods

### 2.1. Sample Collection

The propolis samples were collected from different regions of Balochistan province: Ziarat (P1), Kalat (P2), Sibi (P3), Kohlu (P4), Hub (P5), Bela (P6), Musa Khail (P7), and Jaffar Abad (P8).

### 2.2. Sample Preparation

To avoid direct sun exposure, all propolis samples were dried for 2-3 weeks at room temperature in dark containers. The dried samples were grounded by mechanical grinder to particle size of about 10–80 *μ*m [8].

### 2.3. Maceration Extraction

Dried propolis sample (10 g) was extracted with 100 mL ethanol as extraction solvent following standard procedures described by Akbar et al. [9]. The flask was intermittently shaken for 24 h followed by filtration through Whatman No. 1 filter. The extracts were concentrated with the help of rotary evaporator followed by freeze drying to obtained powdered extract.

### 2.4. Phytochemical Analysis

The presence of phytochemicals such as alkaloids, tannins, cardiac glycosides, anthraquinones, saponins, flavonoids, coumarins, quinones, steroids, terpenoids, and phlobatannins in propolis extracts was determined by using standard protocols [9, 10].

### 2.5. Total Phenolic and Flavonoid Contents

TPC and TFC were determined using colorimetric methods as described by Sadiq et al. [11]. Gallic as was used as a reference standard for TPC, and quercetin was used as reference standard for TFC. Results were expressed as mg of gallic acid equivalent per gram for TPC and mg of quercetin equivalent per gram for TFC.

### 2.6. DPPH Free Radical Scavenging Activity

Antioxidant activity of propolis extract was evaluated by DPPH free radical scavenging ability. Propolis extract (50 *μ*l) was treated with 5 mL of DPPH (40 PPM produced in ethanol), and the reaction mixture was held at room temperature and in the dark for 30 minutes. At 517 nm, the absorbance of the resulting combination was measured. DPPH solution was used as control. DPPH inhibition (%) was calculated by Equation (1):

(1) $$\begin{array}{c} \text{DPPH inhibition}\left(\%\right)=\left[\left(\text{A}1-\text{A}2\right)/\text{A}1\right]\times 100. \end{array}$$

In above equation, *A*1 is the control (DPPH) absorbance, while *A*2 is absorbance of extract. The *IC*_50_ value expresses the antioxidant ability of the extract. The IC_50_ value required for 50% DPPH inhibition was estimated from the relationship curve of scavenging activities against different concentrations of propolis sample.

### 2.7. Fourier Transform Infrared Analysis (FTIR)

The extract was chemically characterized by FTIR spectrometer (Nicolet, Avatar 360). The sample (5 *μ*L) was placed in FTIR and spectrum was recorded in the range of 4000-500 cm^−1^ with resolution of 4 cm^−1^.

### 2.8. Total Protein Analysis by Lowry's Method

The protein content of propolis extract was determined by using the Lowry's method [12]. Briefly, 4.5 mL of reagent 1 (48 mL of 2% sodium carbonate in 0.1 N NaOH +1 mL of 1% KNaC_4_H_4_O_6_.4H_2_O+1 mL of 0.5% CuSO_4_.5H_2_O) was added to 0.5 mL of each extract and incubated for 15 min at room temperature. After that 0.5 mL of freshly prepared reagent 2 (2 mL Folin Ciocalteu reagent, 2 mL distilled water) was mixed rapidly into the mixture and incubated for 30 min in dark. Bovine serum albumin (BSA) was used as a standard for the procedure, and deionized water was used as blank. Subsequently, the absorbance of the standard solutions and sample extract was measured at 660 nm. The quantification was performed in triplicate, and the amount of protein was expressed as mg BSAE g^−1^ of sample [13].

### 2.9. Carbohydrate Analysis

Carbohydrates were estimated by phenol sulfuric reagent method. 0.5 mL of extract was treated with 0.05 mL of 80% phenol followed by 5 mL of concentrated sulfuric acid and allowed to stand for 10 min. The mixture was shaken and placed for 10 to 20 min in a water bath at 25 to 30 °C, and change in characteristic yellow orange color was observed before readings were taken. The absorbance was measured at 510 nm with glucose, used as standard, and deionized water was used as blank. The results were expressed as mg GE g^−1^ [13].

### 2.10. Antibacterial Activity

The propolis extract was evaluated against different bacterial strains (*Escherichia coli*, *Staphylococcus aureus*, *Pseudomonas aeruginosa*, and *Klebsiella pneumoniae*) by agar well diffusion assay. Mueller Hinton Agar (Oxoid, UK) plates were prepared, and the bacterial culture (10^7^ CFU/ml) was spread over the agar plates. Wells were made by 6-mm cork borer in agar plates, and 170 *μ*L of three concentrations (25, 50, and 100 mg mL ^−1^) of each extract were introduced into the agar wells. Doxycycline was used as positive control, whereas dimethyl sulfoxide (DMSO) was used as negative control. Incubation was carried out for 24 h at 37 °C. The results were expressed in terms of diameter of inhibition zone around the wells [14].

### 2.11. Antifungal Activity

For antifungal activity, 2 g of propolis extract and 1 mL DMSO were added to freshly prepared Sabouraud dextrose agar (Oxoid, UK), and after homogenization, 25 mL of the agar was added into each petri plate. After solidification, 6-mm diameter wells were made in agar by cork borer and fungal plugs (*Aspergillus parasiticus*, *Aspergillus niger*, and *Aspergillus flavus)* of same size were inoculated into wells. The antifungal drug fluconazole was used as a reference. Plates were incubated at 37 °C for 72 h, and the diameter of inhibition zone around the well was recorded [14]. Results are calculated by the following equation:

(2) $$\begin{array}{c} \%\text{Inhibition}=100–\text{linear growth in test}\left(\text{mm}\right)/\text{linear growth in control}\left(\text{mm}\right)\times 100. \end{array}$$

### 2.12. Antileishmanial Assay

The antileishmanial activity of propolis extracts was evaluated by following the method [14] with slight variations. Propolis extracts were analyzed against *L. major* (promastigotes) in culture by 96-well plate. Simply, 1 × 10^7^ cells/mL of promastigotes at log phase was used. The promastigotes were grown in NNN biphasic medium, and 1 mg mL^−1^ of the stock solution was prepared in DMSO. A twofold serial dilution of each sample was carried out, and 10 *μ*L of each dilution with 50 *μ*L of the promastigotes log-phase culture was dispensed to each well of a 96-well plate. Glucantime were used as a standard drug. Plates were incubated at 37 °C for 72 hours, after which 1 mL of DMSO was added to each well, and 20 mL of NBT solution (5 mg/mL in phosphate buffer, pH 7.2) was used to confirm the mortality of the test and standard drugs. The *IC*_50_ values were computed using the linear regression approach, and the absorbance was measured using a Microplate Reader (RT-6000) at 630 nm. The percent of cell viability is calculated by using the following equations:

(3) $$\begin{array}{c} \%\text{cell viability}=\text{Absorbance of test sample}/\text{Absorbance of control}\times 100, \end{array}$$

(4) $$\begin{array}{c} \%\text{inhibition}=100-\%\text{viability}, \end{array}$$

### 2.13. MTT Cell Assay

The cytotoxic effect of propolis extracts against HeLa cell line (cervical cancer carcinoma) was determined by using MTT 3-(4,5-dimethylthiazol-2-yl)-2,5-diphenyltetrazolium bromide colorimetric assay as described by Javed et al. [15]. HeLa cell line was cultured at 37 °C in a humidified atmosphere with 5% CO_2_ in minimal essential medium supplemented with 10% FBS, 100 mg mL^−1^ streptomycin and 100 units mL^−1^ penicillin. After that, 180 mL of cell suspensions (1 × 106 cell mL^−1^) were added in 96-well plates and treated with 100 mg mL^−1^ of each extract and incubated for 48 h. By dissolving MTT in phosphate-buffered saline (PBS, pH 7.2), 20 mL (5 mg mL^−1^ in phosphate buffer) of 0.5 percent 3-(4,5-dimethylthiazol-2-yl)-2,5-diphenyltetrazolium bromide (MTT solution) was added, and the mixture was then incubated for 3 hours at 37 °C to determine the viability of the cells. After the incubation period, the supernatant in each well was carefully removed, and 1 mL of DMSO was then added to each well. Utilizing a UV spectrophotometer, absorbance at 570 nm was used to calculate the amount of formazan produced. Doxorubicin (100 mg mL^−1^) served as the standard medication, and DMSO served as the negative control. Measurements were made to determine the concentration necessary for 50% inhibition (*IC*_50_). The percent of cell viability was calculated by using the following equation:

(5) $$\begin{array}{c} \%\text{cell viability}=\text{Absorbance of test sample}/\text{Absorbance of control}\times 100. \end{array}$$

### 2.14. Atomic Absorption Spectrophotometry for Trace Elements

Trace elements Fe, Zn, Mn, Ni, Pb, Cd, and Cr analyses were carried out through atomic absorption Perkin Elmer 3110 spectrophotometer with hollow cathode lamps as radiation source operated at 5 mA at 393 and 279 nm wavelengths and acetylene air flame as fuel. Digestion of each sample was done according to a previously reported protocol [14, 16]. Briefly, 0.5 g powder of each sample was taken, and 8 mL mixture of acids (5 mL nitric acid, 2 mL sulfuric acid, and 1 mL perchloric acid) was added. After 24 h, the mixture was heated for 30 min at 60 °C at 150 °C, further heated at 150 °C for 15 min, and allowed the solution to settle. The digested mixture was then transferred to a 50-mL volumetric flask and filled with distilled water before being filtered through Whatman No. 1 filter paper. After wet digestion, these produced solutions were tested for element detection, and the findings were given in *μ*g g^−1^.

### 2.15. Gas Chromatography-Mass Spectrometry Analysis

The propolis sample was analyzed using a Shimadzu (TQ-8040) series GC-MS system (Tokyo, Japan) equipped with an AOC-20i auto sampler supplied with experimental conditions for the Rxi-5 MS capillary column length = 30 m, id = 0.25 mm, and film thickness = 0.25 mm (Bellefonte PA, USA). A sample of 2 *μ*L was injected with an auto sampler in a split ratio of 10 : 1 and the carrier gas helium at a flow rate of 1 ml/min. The mass spectrum was obtained by electron ionization at 70 eV with a mass scan mode range of 45-500 amu (atomic mass units). The injector temperature was set at 280 °C, and the oven temperature was programmed from 50 °C for 3 min and then increased at the rate of 175 °C at 3 °C/min for 5 min and finally to 200 °C for 5 °C/min for 25 min. The solvent cut time was 2.00 min, and the end GC–MS time was 74 min. By comparing the mass spectrum records of the discovered compounds with those of the NIST 14 and 14s (National Institute of Standards and Technology) Libraries, the identification and composition of the compounds were confirmed. The components of propolis were identified by mass spectrometry using their names, molecular formulas, molecular weights, and structures.

### 2.16. Statistical Analysis

The results of all analyses were carried out in triplicate and the data were presented as the mean ± standard deviation (SD). The inhibitory concentrations (*IC*_50_) were measured by the linear regression method.

## 3. Result

### 3.1. Phytochemical Analysis

Phytochemical analysis of propolis extracts indicated the presence of various phytochemicals such as steroids, carbohydrates, flavonoids, coumarins, cardiac glycosides, quinones, anthraquinones, terpenoids, tannins, and phlobatannins at different levels. In sample P1, all phytochemical constituents were present except tannins, anthraquinones, terpenoids, and cardiac glycosides. Cardiac glycosides were found absent in sample P2. Steroids, carbohydrates, flavonoids, coumarins, cardiac glycosides, quinones, anthraquinones, terpenoids, tannins, and phlobatannins were abundant in P3 and P5, but saponins were lacking in P4, which contained all phytochemicals except tannins. Terpenoids, coumarins, and anthraquinones were not found in P6, although carbohydrates, tannins, flavonoids, and phlobatannins were P7, while P8 was rich in all phytochemicals except flavonoids and coumarins in the current study as presented in Table 1.

### 3.2. Total Flavonoid and Phenolic Contents

In present study TFC was ranged between 0.1546 ± 0.087 and 0.6586 ± 0.329 mg of quercetin g^−1^ of propolis extract. The propolis extract (P1) collected from Ziarat region expressed maximum flavonoid content (0.6586 ± 0.329) and the extract (P6) from Bela region have the lowest content (0.1546 ± 0.087) mg of quercetin g^−1^ of propolis extract presented in (Table 2).

In present study, TPC varied between 2.9343 ± 1.247 and 6.0216 ± 2.873 mg of gallic acid g^−1^ of propolis extract. The propolis extract (P3) collected from Sibi was with maximum phenolic content (6.0216 ± 2.873), and the sample (P5) from Hub region showed lowest content (2.9343 ± 1.247) mg of gallic acid g^−1^, respectively as shown in (Table 2).

### 3.3. DPPH Free Radicals Scavenging Activity

DPPH scavenging ability of propolis extracts was evaluated by their concentrations having 50% inhibition (*IC*_50_) that is the concentration of extract required to scavenge 50% DPPH free radicals. The lower *IC*_50_ values indicated higher antioxidant potential and same for radical scavenging activity. In the present study, an inverse relation between DPPH scavenging activity and *IC*_50_ was found. The extract (P3) from Sibi was seen to have greatest antioxidant activity with smallest *IC*_50_ value of (27.07 ± 0.73 mg mL^−1^), and the sample (P8) collected from Jaffar Abad with highest *IC*_50_ value (84.43 ± 2.07 mg mL^−1^) showed lowest antioxidant potential as presented in (Table 3).

### 3.4. FTIR Analysis

FTIR was used to determine the presence of functional groups in propolis extract. Functional groups were examined according to the peaks in spectra [17]. The most stable peaks in the spectrum were at 3734-3648 cm^−1^, and designated to elongation of O-H of hydroxyl bonds and N-H of amino acids, the peaks at 3338-3334 cm^−1^ were given to the O-H stretching of phenolic compounds, and peaks at 3000-3200 cm^−1^ were assigned to the C-H stretching of flavonoids and aromatic rings. Peaks at 2973-2833 cm^−1^ were associated with methylene asymmetric stretching, and the peaks at approximately 2721-2075 cm^−1^ designated to the hydrocarbons' symmetric stretching. The peaks at 1683-1636 cm^−1^ were assigned to C=O, C=C stretching vibrations of flavonoids and designated to the N-H asymmetric stretching of amino acids. In addition, there was a high correspondence of the signals at 1558-1506 cm^−1^; ascribed to elongation of flavonoids and aromatic rings, the peak at 1456-1400 cm^−1^ were associated with bending vibrations C-H, CH_2_, and CH_3_ of flavonoid and aromatic rings. The main characteristics of extract was explained from the signals of stretching vibrations and bending at 1399-1310 cm^−1^ and 1230-1203 cm^−1^ attributed to asymmetrical O-H and C-CO bending of hydrocarbons and phenol groups. The peaks at 1198-1000 cm^−1^ were designated to stretching vibrations of C-C of flavonoids and secondary alcohol groups. Specifically, a symmetrical stretching at 945-881 cm^−1^ linked to C-C-O, primary, and secondary alcohols. The FTIR results confirmed the presence of hydrocarbons, flavonoids, aromatic compounds, phenolic compounds, primary and secondary alcohols, and amino acids as presented in (Table 4).

### 3.5. Total Protein Content

Protein content in propolis extract was determined by using Lowery's method. The total proteins content ranged from 0.018 ± 0.020 to 0.834 ± 0.282 mg of BSAE g^−1^ of propolis extract. Sample P3 showed maximum protein content 0.834 ± 0.282, whereas the minimum protein content was found in P8 as 0.018 ± 0.020 mg of BSA g^−1^ of propolis extract as presented in (Table 5).

### 3.6. Total Carbohydrates Content

The results of carbohydrate content in propolis extract ranged from 0.356 ± 0.066 to 3.616 ± 0.802 mg of glucose g^−1^ of propolis extract. The highest content of carbohydrate found in (P1) as 3.616 ± 0.802 mg/g, whereas the lowest content was found in P5 as 0.356 ± 0.066 mg/g, respectively. Results are shown in (Table 5).

### 3.7. Antibacterial Activity

Propolis extract (P3) showed maximum zone of inhibition against *E. coli* (19.06 ± 1.90 mm) followed by *S. aureus* with the diameter of inhibition zone of 16.73 ± 2.01 mm, *P. aeruginosa*15.73 ± 1.41 mm, and *K. pneumoniae*14.66 ± 2.51 mm, respectively. The propolis extract (P4) collected from Kohlu was highly active against *K. pneumoniae* than other gram-positive and gram-negative bacteria with the maximum diameter of zone of inhibition 20.33 ± 1.52 mm. Propolis extract (P2) was found less active against S. *aureus* and *E. coli* with the diameter of inhibition zone of 7.44 and 8.66 mm, respectively (Table 6).

### 3.8. Antifungal Activity

The antifungal activity of propolis extracts was analyzed against three fungal species *A. parasiticus*, *A. niger*, and *A. flavus*. *A. niger* was highly sensitive to propolis extract (P3) with inhibition of 82% followed by *A.s flavus* with 81%, while *A. parasiticus* showed the 79% inhibition, respectively. Among other samples, propolis extract (P1) was highly active against *A. parasiticus* with 80% of inhibition (Table 7).

### 3.9. Antileishmanial Assay

**Figure 1:**
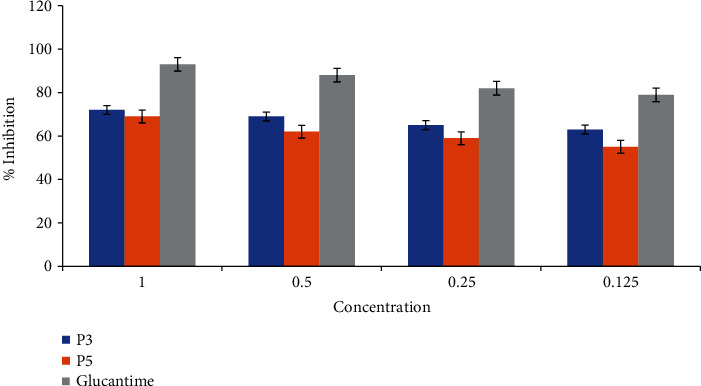
Efficiency of ethanolic extracts of propolis P3 and P5 against promastigotes (*Leishmania major*).

For formative antileishmanial activity of promastigotes (*L. major*), antileishmanial assay was performed against two propolis samples P3 and P5. A twofold serial dilution of each sample (1 mg mL^−1^) was carried out. The standard drug Glucantime (*IC*_50_ = 7.31 ± 0.64 mg mL^−1^) was used to compare the parasite inhibition with each extract. The *IC*_50_ value was observed for both extract against *L. major*, and P5 showed good potential (*IC*_50_ = 11.25 ± 1.09 mg mL^−1^), followed by the P3 (*IC*_50_ = 16.35 ± 0.26 mg mL^−1^) by comparing the values of each extract with the standard. The results revealed that all concentrations showed highest % inhibition and the viability increased with a decrease in concentration, as presented in Figure 1.

### 3.10. MTT Cell Assay

**Figure 2:**
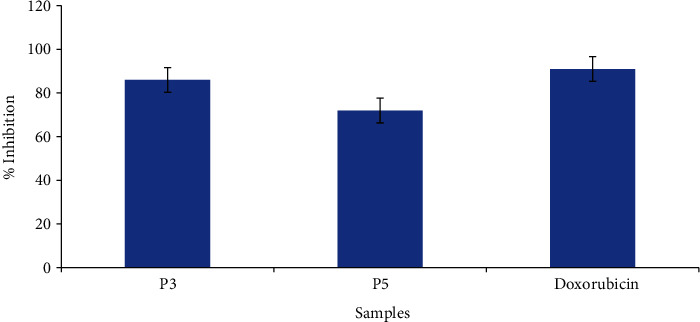
Anticancer activity of propolis extracts P3 and P5 against HeLa cell line. Bars represent the standard deviation of the mean.

Evaluation of the anticancer activity of propolis extracts was carried out through MTT cell assay against HeLa cell line. The assay was performed at 100 mg mL^−1^ for each extract by using doxorubicin as a standard drug. According to the obtained results, each extract exhibited anticancer activity, and % inhibition was expressed in terms of *IC*_50_. Comparing the results to the standard (*IC*_50_ 11 ± 0.32 mg mL^−1^), it was revealed that the propolis extract (P3) showed the highest anticancer activity with lowest *IC*_50_ (15 ± 0.26 mg mL^−1^) followed by P5 having *IC*_50_ value of 19 ± 0.12 mg mL^−1^, respectively, (Figure 2).

### 3.11. Atomic Absorption Spectrophotometer of Trace Elements

For the determination of trace elements, different propolis samples P1-P8 were analyzed by atomic absorption Perkin Elmer 3110 spectrophotometer. According to the obtained results, Fe was 443.38 ± 0.3 *μ*g g^−1^ in P1, 534.67 ± 0.2 *μ*g g^−1^ in P2, 823.84 ± 0.1 *μ*g g^−1^ in P3, 355.17 ± 0.7 *μ*g g^−^ in P4, 528.96 ± 0.1 *μ*g g^−1^ in P5, 1331.46 ± 0.5 *μ*g g^−1^ in P6, 1079.68 ± 0.1 *μ*g g^−1^ in P7, and 663.31 ± 0.4 *μ*g g^−1^ in P8, respectively. Concentration of Zn was in range of 0.257 ± 0.5 *μ*g g^−1^ in P1 to 0.472 ± 1.2 *μ*g g^−1^ in P2. Concentration of Mn varied in the range of 040.86 ± 0.8 *μ*g g^−1^ in P5 to 077.44 ± 0.6 *μ*g g^−1^in P3. Among all samples, lower Ni concentration was 1.25 ± 0.16 *μ*g g^−1^ in P2, and higher was 64.23 ± 0.4 *μ*g g^−1^ in P7. Pb was 4.58 ± 1.2 *μ*g g^−1^ in P6 to 10.50 ± 0.6 *μ*g g^−1^ in P7, respectively. Higher concentration of Cd was 0.412 ± 0.5 *μ*g g^−1^ in P4, and lower concentration was 0.010 ± 0.2 *μ*g g^−1^ present in P7. Cr was present in very less amount in all evaluated samples. Minimum concentration was 0.001 ± 0.4 *μ*g g^−1^ in P5, and maximum concentration was 0.115 ± 0.3 *μ*g g^−1^ in P1, respectively, as presented in (Table 8).

### 3.12. GC-MS Analysis

The results of the GC-MS analysis showed that a total of 80 different compounds were identified. These compounds belonged to various chemical classes. Results of identified compounds were presented in terms of their retention time, concentration (area %), molecular formula, and molecular weight as shown in Table 9. The identified compound belonged to ethers, alcohols, terpenes, phenolics, acids, and other aromatic compounds.

## 4. Discussion

The current study was aimed to analyze the bioactive components, antimicrobial activity, and the presence of trace elements of propolis samples collected from different areas of Balochistan. Phytochemical analysis is critical for identifying bioactive molecules that may lead to medication development and discovery. Propolis contains a wide variety of secondary metabolites such as steroids, flavonoids, tannins, alkaloids, and terpenoids, which have antibacterial, antitumor, anthelmintic, anti-inflammatory, and antiradical activities [18]. The ethanolic extract of propolis obtained from different parts of Balochistan contained almost all the components, tannins, cardiac glycosides, saponins, terpenoids, flavonoids, coumarin, quinones, phlobatannins, and anthraquinones. The intensity of the color may indicate a higher concentration of these compounds in propolis extract. The ethanolic extract of Malaysian propolis has been found to contain flavonoids, alkaloids, cardiac glycosides, tannins, saponins, phenol, xanthoproteins, terpenoids, and resins [19]. Variations in propolis composition are highly influenced due to phytogeographical diversity, climate change, seasonal variations, and specie of the queen bee [1].

Polyphenols are leading group of phytochemicals and deemed as active component of propolis. These compounds comprise are good reducing agents due to which they act as good antioxidants. The results concerning TPC indicated variations among all extracts. The highest TPC value 6.0216 ± 2.873 mg of gallic acid g^−1^ was found in P3 extract whereas minimum value 2.9343 ± 1.247 mg of gallic acid g^−1^ was obtained in the sample P5 as shown in (Table 2). The results of total phenolic contents of ethanolic extracts were comparatively minimum than other reported studies [3] and supported by the previously observations of [1] who evaluated the total phenolic contents of propolis collected from peripheral region of Faisalabad Pakistan. Results are also in accordance with [20] in propolis samples collected from different regions of Korea.

Flavonoid contents are known to have antimicrobial, antioxidant, anti-inflammatory, and antidepressant potential. These contents are good antioxidants having strong reducing potentials. In present study, total flavonoid content was ranged between 0.1546 ± 0.087 and 0.6586 ± 0.329 mg of quercetin g^−1^ of propolis extract. The propolis extract (P1) collected from Ziarat region expressed maximum flavonoid content (0.6586 ± 0.329) and the extract (P6) from Bela region have the lowest content (0.1546 ± 0.087) mg of quercetin g^−1^ of propolis extract presented in (Table 2). Results of current study are minimum than other reported studies from different location in Turkey [3], Pakistan [1], China [21], and Iran [22]. According to Choi et al. [20], the variations in total phenolic and total flavonoids of propolis samples depend on their geographic origin.

The DPPH assay for analyzing free radical scavenging activity is widely accepted feature for the evaluation of antioxidant potential of natural extracts. The antioxidant ability of propolis extracts of different regions of Balochistan were analyzed by their concentrations having 50% inhibition (*IC*_50_) that is the concentration of extract required to scavenge 50% DPPH free radicals. The lower *IC*_50_ values indicated higher antioxidant potential and same for radical scavenging activity. In present study, an inverse relation between DPPH scavenging activity and *IC*_50_ was found. The extract (P3) from Sibi was seen to have greatest antioxidant activity with smallest *IC*_50_ value of (27.07 ± 0.73 mg mL^−1^), and the sample (P8) collected from Jaffar Abad with highest *IC*_50_ value (94.43 ± 2.07 mg mL^−1^) showed lowest antioxidant potential as presented in (Table 3). According to Zehra, Yildiz, Şahin, Asadov, and Kolayli [23], the antioxidant potential of propolis extracts have a positive correlation with their phenolic, flavonoids, and other bioactive contents. Recentlym Shahbaz et al. [1] reported the DPPH free radical scavenging activity of propolis up to 70% which is in accord to the findings of current study. In a similar way Choi et al. [20] concluded that propolis extract from Korea exhibits higher antioxidant activity as compared to Brazilian propolis due to higher concentration of flavonoids and phenolic contents. Furthermore, Al-Juhaimi et al. [3] concluded that DPPH inhibition is the direct function of phenolic contents present in samples. Current results for the percent inhibition of DPPH were in agreement with their findings.

The FTIR analysis of different propolis extract was carried out to characterize functional groups present in samples. The FTIR results confirmed the presence of hydrocarbons, flavonoids, aromatic compounds, phenolic compounds, primary and secondary alcohols, and amino acids. According to Ahmed, Amirat, Aissat, Aissa, and Khiati [24], the propolis' FTIR data revealed the presence of O-H stretch and C-H bound for alcohol at frequencies between 2848 cm^−1^ and 2915 cm^−1^, as well as O-H and C=O at 1168 cm^−1^ and C-O and C-C at 1000 cm^−1^, respectively. The OH group can be seen in FTIR spectra between 3550 and 3540 cm^1^, and an asymmetric CH2 methyl group can be seen at 2900 cm^1^, according to a recent publication [17]. The geochemistry of the soil where propolis is grown may have a significant impact on the content and components of propolis.

Protein content in propolis extract was determined by using Lowery's method. The total proteins content was ranged from 0.018 ± 0.020 to 0.834 ± 0.282 mg of BSAE g^−1^ of propolis extract. Sample P3 showed maximum protein content 0.834 ± 0.282, whereas the minimum protein content was found in P8 as 0.018 ± 0.020 mg of BSAg^−1^ of propolis extract. Total protein contents were in agreement with the values reported by Laaroussi et al. [25] with revealed values of 1.65% to 6.18%, respectively. Current results were also in accordance with findings of [26] for propolis from different geographic regions. The presence of protein in propolis is mostly related to the pollen fraction added by bees for bee glue production.

Carbohydrates are one of the three macronutrients used in diet, along with protein. The total carbohydrates in propolis extract were estimated using the phenol sulfuric technique. The carbohydrate content of propolis extract in our study ranged from 0.356 ± 0.066 mg of glucose g^−1^ to 3.616 ± 0.802 mg of glucose g^−1^. The highest carbohydrate content was identified in P1 at 3.616 ± 0.802, while the lowest was found in P5 at 0.356 ± 0.066. Current findings are in agreement with the values reported by Laaroussi et al. [25]. According to Fikri, Popova, Sulaeman, and Bankova [27], the harvesting techniques of propolis may influence the carbohydrate contents due to sugar residues from honey. Additionally, buds are the potential sources of carbohydrates in propolis.

Rich in polyphenols and flavonoids, propolis has excellent antibacterial power against pathogenic germs without having any negative effects. Propolis antimicrobial properties are extremely significant for the bee colony. By interfering with the enzymatic activity of bacteria, propolis prevents the growth of bacteria. Propolis can harm both gram-positive and gram-negative bacteria. The propolis' primary ingredients, phenols, flavonoids, phenolic acids, and their esters, are what give it its potent antibacterial activity [28]. In current study propolis showed antibacterial activity against *S. aureus*, *E. coli*, *K. pneumonia*, and *P. aeruginosa*. Propolis extract (P3) showed maximum zone of inhibition against *E. coli*19.06 ± 1.90 followed by *S. aureus* with the diameter of inhibition zone of 16.73 ± 2.01, *P. aeruginosa*15.73 ± 1.41, and *K. pneumoniae*14.66 ± 2.51 mm, respectively. The propolis extract (P4) collected from Sibi was highly active against *K. pneumoniae* than other gram-positive and gram-negative bacteria with the maximum diameter of zone of inhibition 20.33 ± 1.52 mm, while propolis extract (P2) was found less active with minimum diameter of zone of inhibition 7.46 ± 1.50 mm followed by *E. coli* with the diameter of inhibition zone of 8.66 ± 0.28 mm, respectively. The antibacterial effects of propolis results on *S. aureus* and *Escherichia coli* are in agreement with Shahbaz et al. [1] and relatively higher than Afata et al. [18]. Studies have linked propolis from Brazil, Egypt, Mangolia, and Albania to antibacterial activity against *S. aureus*, with zones of inhibition of 24, 21.8, 24.3, and 21.8 mm, respectively [1]. Different extraction techniques very certainly produce different chemical components, which could ultimately cause variations in the antibacterial activity [29]. It is unclear if the antibacterial effect is brought on by a single active component or by the combination of several active elements found in propolis extracts. However, Al-Juhaimi et al. [3] concluded that propolis strength against bacterial strains may be due to the strong effect of phenolics, flavonoids ,and other components present in propolis extracts.

The antifungal activity of propolis extracts was tested in terms of the % age of inhibition zone against three filamentous fungal species *A. parasiticus*, *A. niger*, and *A. flavus*. All extracts were found highly active against all three fungi. *Aspergillus Niger* was highly sensitive to propolis extract (P3) with the percentage of zone of inhibition 82% followed by *A. flavus* with 81%, while *A. parasiticus* showed the percentage of zone of inhibition 79%, respectively. Among other samples, propolis extract (P1) was highly active against *A. parasiticus* with 80% of zone of inhibition. The great potential for antifungal activity and trend was found in consistent with the literature [30, 31]. However, the present findings were relatively higher than Afata et al. [18], where extracts showed minimum results against *Aspergillus niger.* The antifungal activity of each extract may be due to the presence of antifungal compounds that include linalool and other phenolic and flavonoid compounds reported in different propolis extracts [32].

Efficiency of ethanolic extracts of propolis P3 and P5 against promastigotes (*Leishmania major*) was determined by antileishmanial assay. A twofold serial dilution of (1 mg mL^−1^) sample was carried out, and the activity was checked at different concentrations (1, 0.5, 0.25, and 0.125 mg mL^−1^) against standard drug Glucantime proven by *IC*_50_ values (*IC*_50_ 7.31 ± 0.64 mg mL^−1^). The activity was carried out under an incubation period of 48 h at 22 °C. Moreover, 50% inhibitory concentration was observed for each extract and the P5 showed good potential (*IC*_50_ 11.25 ± 1.09 mg mL^−1^), followed by P3 (*IC*_50_ 16.35 ± 0.26 mg mL^−1^). The results revealed that the highest activity was observed in the concentration of (1 mg mL^−1^) and the viability increased with a decrease in concentration and the %inhibition decreased. Propolis extracts have been evaluated against leishmanial parasites from different part of the world and have proven with significant leishmanicidal potentials. According to Do Nascimento et al. [33], the ethanolic extract of Brazilian red propolis showed *IC*_50_ of 37.9 *μ*g mL^−1^ and nanoparticles of red propolis extract *IC*_50_ of 31.34 *μ*g mL^−1^. Previously, Duran, Muz, Culha, Duran, and Ozer [34] analyzed antileishmanial activity of Turkey propolis with excellent leishmanicidal effect *IC*_50_ of 250 and 500 *μ*g mL^−1^. In another study, Brazilian propolis extract showed *IC*_50_ 49 *μ*g mL^−1^ against *L. major* species, while the Bulgarian propolis extract showed leishmanicidal activity for *L. chagasi* and *L. major* species with IC_50_ 2.8 to 41.3 *μ*g mL^−1^. According to scientific literature, excellent leishmanicidal activity of propolis can be explained by the presence of various flavonoids, such as quercetin, fisetin, and luteolin, and some phenolic acids and phenolic acid esters in the extracts [33]. Description of the literature demonstrated that some flavonoids have leishmanicidal effects with *IC*_50_ values from 0.6 to 0.8 *μ*g mL^−1^.

Cancer is one of the major diseases that are enlightened by increasing the human body cells in with the failure to be controlled. Therefore, various studies have been reported to develop new therapeutic treatments for cancer. Propolis is a rich source of biologically potent compounds regulating several cellular processes. The anticancer activity of propolis from various regions of Balochistan Pakistan have not been reported and published by other researchers until date, but researchers from different parts of the world reported propolis to have in cytotoxic effects against different destructive cell lines. According to Forma and Bryś [4], both propolis extracts and active chemicals can decrease cancer cell growth, angiogenesis, and metastasis while also stimulating apoptosis. It may potentially have an impact on cancer multidrug resistance. Few studies reported the strong cytotoxic activity of galangin, syringic acid, caffeic acid, and ferulic acid against different cancer cell lines [35]. In vitro, an ethanolic extract of Algerian propolis and galangin reduced the number of B16F1 melanoma cells compared to a reference [36]. Recently, Fang, Xiong, Xu, Yin, and Luo [37] reported the proapoptotic activity of polyphenolic compounds such as ferulic acid and caffeic acid on human tongue squamous carcinoma cells (CAL-27). Current results were higher than the findings of Dastan et al. [22] who reported propolis methanolic extracts with (*IC*_50_) 702.5 and 177.7 *μ*g mL^−1^ after 24 and 48 hours.

The AAS is a method of analysis that provides the estimated concentration of different elements. Different samples P1, P2, P3, P4, P5, P6, P7, and P8 were examined for the content of two nonessentials (lead and cadmium) and five essential elements (iron, zinc, nickel, chromium, and manganese).

The human body requires iron for the synthesis of oxygen to produce red blood cells. Anemia is brought on by Fe deficiency, but excessive amounts harm body tissues. In general, iron is not thought to have negative health effects unless it is consumed in excessively high doses [14, 16]. The currents findings were ranged from 355.17 *μ*g g^−1^ in P11 to 1331.46 *μ*g g^−1^ in P13, relatively higher than previous findings [38].

Zinc is second most prevailing transition metal in organisms after iron. It promotes the carbon incorporation and terpene consumption and antioxidant enzyme activation. The concentration of Zn was in range of 0.257 ± 0.5 *μ*g g^−1^ in P1 to 0.472 ± 1.2 *μ*g g^−1^ in P2. Manganese is good antioxidant and important for plant and animal growth. It is a low toxic element with considerable biological application, and its deficiency causes reproductive problems in mammals, and excessive amount leads to different lungs and brain diseases [14, 16]. It helps in the synthesis and activation of many enzymes. The Mn concentration in present work varied in the range of 40.86 *μ*g g^−1^ in P10 to 77.44 *μ*g g^−1^ in P12. Current results were relatively higher than previous findings [38]. Nickel is an important element that controls different metabolic processes in plants. Nickel is present in RNA and DNA of human body where it functions in association with nucleic acids. Nickel shows carcinogenic side effects when taken in higher concentrations; however, its deficiency causes heart and liver problems [14, 16]. Among all samples, lower Ni concentration was −5.92 ± 0.13 *μ*g g^−1^ in P1 and higher was 64.23 ± 0.4 *μ*g g^−1^ in P7, respectively. Current results were in accordance with previous findings [38] and higher than Zeb et al. [39]. Cadmium concentration may occur in bee products from air and mineral fertilizers, and its presence in certain concentrations in organisms can have adverse effects [40]. This toxic element can damage the brain, kidney, liver, and heart. In present work the concentration of Cd varied from 0.010 ± 0.2 *μ*g g^−1^ present in P7 to 0.412 ± 0.5 *μ*g g^−1^ in P4. Various studies conducted have shown Cd concentration in propolis samples [38]. However, present results were relatively higher than the permissible limit and those found in literature [39]. Long term exposure to Pb can cause severe health effects such as chronic pain, blood pressure alteration, and change in blood composition, anxiety, passivity disorders, and cancer. According to the current findings Pb was ranged from 4.58 ± 1.2 *μ*g g^−1^ in P6 to 10.50 ± 0.6 *μ*g g^−1^ in P7, respectively. Vehicular emission on the nearby roadway and use of fertilizers are the most important explanation for the high Pb concentration. Various studies conducted have shown Pb concentration in various propolis samples [38, 40]. Chromium was present in very less amount in all evaluated samples. Minimum concentration of Cr was 0.001 ± 0.4 *μ*g g^−1^ in P5, and maximum concentration was 0.115 ± 0.3 *μ*g g^−1^ in P1. Chromium concentration in all evaluated samples was less than the permissible limit. Current findings were in agreement with Achudume and Nwafor [41] and lower than the results of Ullah et al. [38].

The results of the GC-MS (gas chromatography-mass spectrometry) analysis showed that a total of 80 different compounds were present in propolis. These compounds belonged to various chemical classes such as aromatic acids, esters, alcohols, flavonoids, and terpenes. Accordingly, it is believed that the presence of flavonoids [42] may cause the antibacterial and cytotoxic effects. The compounds, hexadecanoic acid, methyl ester, 9,12-Octadecadienoic acid (Z,Z)-, and methyl ester are previously been reported for their antioxidant, anti-inflammatory, cytotoxic, and antibacterial potentials (Fahad et al., 2021).

## 5. Conclusion

Present study represents the data about chemical composition of propolis collected from different regions of Balochistan. Overall, the results of this report show that propolis is rich in phenolic and flavonoid contents with high antioxidant potentials. The use of propolis as an active agent for the treatment of various infectious diseases could also be supported by the strength of broad spectrum antibacterial and antifungal activities. Moreover, all analyzed samples revealed a great variation in their trace elements. However, more research is necessary to publicize the biological activity of the identified bioactive components and their therapeutic potential.

## Figures and Tables

**Table 1:** Phytochemical analysis of propolis collected from different sites.

S. no	Phytochemical test	P1	P2	P3	P4	P5	P6	P7	P8
1.	Carbohydrates	+	+	+	+	+	+	−	+
2.	Cardiac glycosides	−	−	+	+	+	+	+	+
3.	Tannins	−	+	+	−	+	+	−	+
4.	Steroids	+	+	+	+	+	+	+	+
5.	Terpenoids	−	+	+	+	+	−	+	+
6.	Flavonoids	+	+	+	+	+	+	−	−
7.	Saponins	+	+	−	+	−	+	+	+
8.	Coumarin	+	+	+	+	+	−	+	−
9.	Quinones	+	+	−	+	+	+	+	+
10.	Anthraquinones	−	+	+	+	+	−	+	+
11.	Phlobatannins	+	+	+	+	+	+	−	+

Note: (+) sign indicates the presence of phytochemicals, while (−) sign indicates the absence.

**Table 2:** Total flavonoid content and total phenolic content of different propolis extracts.

Sample	Total flavonoid content (mg QE g^−1^) ± SD	Total phenolic content (mg GAE g^−1^) ± SD
P1	0.6586 ± 0.329	5.9209 ± 2.880
P2	0.6025 ± 0.339	4.1074 ± 3.445
P3	0.3873 ± 0.543	6.0216 ± 2.873
P4	0.5923 ± 0.297	5.7347 ± 4.099
P5	0.4291 ± 0.449	2.9343 ± 1.247
P6	0.1546 ± 0.087	5.9194 ± 3.247
P7	0.5636 ± 0.399	5.0588 ± 1.005
P8	0.6467 ± 0.305	4.9128 ± 2.589

Results are expressed as mean of three determinations ± standard deviation.

**Table 3:** DPPH free radicals scavenging activity of propolis extracts and *IC*_50_ (mg mL^−1^).

Samples	DPPH% scavenging activity	*IC*_50_ mg mL^−1^
P1	56.81 ± 3.08	79.09 ± 0.91
P2	66.43 ± 4.37	41.04 ± 1.28
P3	68.39 ± 1.02	27.07 ± 0.73
P4	64.19 ± 09.5	54.75 ± 0.97
P5	64.44 ± 3.26	52.53 ± 1.16
P6	61.64 ± 2.13	60.81 ± 0.65
P7	58.52 ± 2.08	77.03 ± 1.04
P8	52.79 ± 1.09	84.43 ± 2.07

Results are expressed as mean of three determinations ± standard deviation.

**Table 4:** Fourier Transform infrared analysis of propolis extract.

Range (cm^−1^)	Type of signal	Type of link	Main attribution
3740-3550	Elongation	O-H and N-H	Hydroxyls and amino acids
3366-3333	Stretching	O-H	Phenolic groups
3000-3200	Stretching	C-H, aromatics	Flavonoids and aromatic rings
2971-2830 and 2730-2066	Elongation symmetric and asymmetric	C-H	Hydrocarbons
1699-1610	Asymmetric bending vibrations	C=O	Lipids, flavonoids and amino acids
1560-1505	Elongation	C=C, aromatics	Flavonoids and aromatic rings
1450-1415	Bending vibrations	C-H, C-H_2_ and C-H_3_	Flavonoids and aromatic rings
1399-1310	Bending vibration	C-H	CH3 group of flavonoids
1232-1200	Bending vibration (O-H) and asymmetrically bending (C-CO)	O-H and C-CO	Hydrocarbons
1198-1000	Stretching vibration (C-C) and bending (C-OH)	C-C and C-OH	Flavonoids and secondary alcohol groups

**Table 5:** Estimation of total protein content and total carbohydrate content.

Samples	Total proteins (mg BSAE g^−1^) ± SD	Total carbohydrate (mg GE g^−1^) ± SD
P1	0.478 ± 0.143	3.616 ± 0.802
P2	0.316 ± 0.184	0.377 ± 0.124
P3	0.834 ± 0.282	0.554 ± 0.080
P4	0.765 ± 0.266	0.498 ± 0.787
P5	0.282 ± 0.363	0.356 ± 0.066
P6	0.663 ± 0.220	0.629 ± 0.106
P7	0.442 ± 0.112	0.598 ± 0.063
P8	0.018 ± 0.020	0.504 ± 0.068

Results are expressed as mean ± SD for three readings.

**Table 6:** Antibacterial activity of propolis extract against pathogenic bacteria.

Samples	Diameter of zone of inhibition against pathogen			
	*E. coli*	*S. aureus*	*K. pneumoniae*	*P. aeruginosa*
P1	14.3 ± 1.50	13.8 ± 1.25	13.8 ± 3.01	16.5 ± 1.80
P2	8.66 ± 0.28	7.46 ± 1.50	12.83 ± 2.25	12.73 ± 1.55
P3	19.06 ± 1.90	16.73 ± 2.01	14.66 ± 2.51	15.73 ± 1.41
P4	12.5 ± 1.32	13.66 ± 2.51	20.33 ± 1.52	11.83 ± 2.84
P5	10.83 ± 1.25	12.1 ± 0.85	11.5 ± 1.5	12.5 ± 1.5
P6	18.5 ± 1.5	14.83 ± 2.56	17.5 ± 1.5	10.15 ± 1.32
P7	10.5 ± 2.29	11.76 ± 1.36	16.76 ± 2.54	10.86 ± 1.20
P8	13.6 ± 1.50	13.83 ± 1.25	12.73 ± 2.96	15.1 ± 0.85
Doxycycline	20.4 ± 1.51	23.8 ± 1.73	23.4 ± 2.14	23.5 ± 1.53

Note. Inhibitory zones in mm as mean ± standard deviation of three replicates.

**Table 7:** Antifungal activity (% inhibition) of propolis extracts against pathogenic fungi.

Samples	*A. parasiticus*	*A. niger*	*A. flavus*
P1	80	65	69
P2	75	71	74
P3	79	82	81
P4	34	50	65
P5	38	46	52
P6	71	69	73
P7	64	67	63
P8	76	52	68
Fluconazole	92	89	94

Results are expressed as % age of inhibition zone against *Aspergillus parasiticus*, *Aspergillus niger*, and *Aspergillus flavus.*

**Table 8:** Atomic absorption spectrophotometry of trace elements found in propolis extract.

Elements	P1 (*μ*g g^−1^)	P2 (*μ*g g^−1^)	P3 (*μ*g g^−1^)	P4 (*μ*g g^−1^)	P5 (*μ*g g^−1^)	P6 (*μ*g g^−1^)	P7 (*μ*g g^−1^)	P8 (*μ*g g^−1^)
Fe	443.38 ± 0.3	534.61 ± 0.2	823.84 ± 0.1	355.17 ± 0.7	528.96 ± 0.1	1331.46 ± 0.5	1079.68 ± 0.1	663.31 ± 0.4
Zn	0.257 ± 0.5	0.472 ± 1.2	0.445 ± 0.3	0.290 ± 0.12	0.450 ± 0.8	0.378 ± 1	0.462 ± 0.12	0.297 ± 0.1
Mn	042.91 ± 0.12	055.96 ± 0.13	077.44 ± 0.6	044.53 ± 0.1	040.86 ± 0.8	052.28 ± 0.3	070.60 ± 0.3	052.17 ± 0.2
Ni	5.92 ± 0.13	1.25 ± 0.16	7.97 ± 0.3	7.93 ± 0.1	13.19 ± 0.5	23.14 ± 0.2	64.23 ± 0.4	26.23 ± 0.7
Pb	6.57 ± 0.12	7.37 ± 0.8	5.78 ± 0.12	9.62 ± 0.8	5.42 ± 0.12	4.58 ± 1.2	10.50 ± 0.6	8.66 ± 0.7
Cd	0.312 ± 0.7	0.217 ± 0.1	0.108 ± 0.1	0.412 ± 0.5	0.112 ± 0.5	0.023 ± 1	0.010 ± 0.2	0.221 ± 0.6
Cr	0.015 ± 0.3	0.007 ± 0.13	0.009 ± 0.5	0.013 ± 0.3	0.001 ± 0.4	0.002 ± 0.6	0.002 ± 0.1	0.006 ± 0.6

Note. Fe = iron; Zn = zinc; Mn = manganese; Ni = nickel; Pb = lead; Cd = cadmium; and Cr = chromium.

**Table 9:** The major compounds analyzed in ethanolic extract of propolis by GCMS analysis.

S. #	Retention time (min)	Area (%)	Name of the compound	Mol. Formula	Mol. Weight
1.	3.035	0.00	2-Chloroethyl methyl sulfoxide	C_3_H_7_ClOS	126
2.	3.083	0.01	Carbonochloridic acid, ethyl ester	C_3_H_5_ClO_2_	108
3.	3.145	0.02	Acetic acid, mercapto-, methyl ester	C_3_H_6_O_2_S	106
4.	3.175	0.01	Propyl mercaptan	C_3_H_8_S	76
5.	3.305	0.01	Dimethyl sulfoxide	C_2_H_6_OS	78
6.	3.401	0.02	Cyclohexan-1,4,5-triol-3-one-1-carboxylic acid	C_7_H_10_O_6_	190
7.	3.425	0.03	S-methyl methanethiosulfonate	C_2_H_6_OS_2_	110
8.	3.469	0.02	o,S,S′-Trimethyl phosphorotrithioate	C_3_H_9_OPS_3_	188
9.	3.579	0.10	Dichloromethylpho sphonic acid	CH_3_Cl_2_O_3_P	164
10.	3.706	0.10	Sulfide, ethyl propyl	C_5_H_12_S	104
11.	3.810	0.05	Carbamimidoylsulfanylacetic acid	C_3_H_6_N_2_O_2_S	134
12.	3.860	0.09	Ethane, 1-chloro-1-fluoro-	C_2_H_4_ClF	82
13.	3.905	0.05	Propane, 1,1,1,2-tetrachloro-	C_3_H_4_Cl_4_	180
14.	3.990	0.06	1,6-Dideoxy-l-mannitol	C_6_H_14_O_4_	150
15.	4.100	0.14	1,3-Difluoro-2-propanol	C_3_H_6_F_2_O	96
16.	4.195	0.10	Diethoxymethyl acetate	C_7_H_14_O_4_	162
17.	4.361	0.16	Silane, bis(fluoromethyl)dimethyl-	C_4_H_10_F_2_Si	142
18.	4.400	0.12	Methoxyacetaldehyde diethyl acetal	C_7_H_16_O_3_	148
19.	4.510	0.12	2-propanol, 1-methoxy-	C_4_H_10_O_2_	90
20.	4.560	0.21	Diethyl pyrocarbonate	C_6_H_10_O_5_	162
21.	6.717	5.87	Ethyl fluoroformate	C_3_H_5_FO_2_	92
22.	7.214	0.21	Glycerin	C_3_H_8_O_3_	92
23.	7.592	0.01	1,2-Propanediol, 1-acetate	C_5_H_10_O_3_	118
24.	7.908	0.03	p-Dioxane-2,3-diol	C_4_H_8_O_4_	120
25.	7.950	0.10	Fluoroacetic acid	C_2_H_3_FO_2_	78
26.	7.987	0.05	Propanoic acid, 2-hydroxy-, methyl ester,	C_4_H_8_O_3_	104
27.	8.071	0.03	1-butanol, 2-nitro-	C_4_H_9_NO_3_	119
28.	8.216	0.05	2-Butenal, 2-methyl-, (E)-	C_5_H_8_O	84
29.	8.366	0.13	Butyl lactate	C_7_H_14_O_3_	146
30.	8.424	0.04	2,3-Butanediol, [R-(R∗,R∗)]-	C_4_H_10_O_2_	90
31.	8.685	0.01	2-Mercaptopropanoic acid	C_3_H_6_O_2_S	106
32.	8.712	0.01	Ethanethiol, 2-(diethylboryloxy)-	C_6_H_15_BOS	146
33.	8.750	0.01	Butanoic acid, 4-chloro-3-oxo-, methyl ester	C_5_H_7_ClO_3_	150
34.	8.853	0.05	3-Cyclopentene-1-acetaldehyde, 2-oxo-	C_7_H_8_O_2_	124
35.	8.970	0.01	1-Nitro-2-acetamido-1,2-dideoxy-d-glucitol	C_8_H_16_N_2_O_7_	252
36.	9.156	0.04	2-Furanmethanol	C_5_H_6_O_2_	98
37.	9.325	0.02	4,5-Dihydro-2-methylimidazole-4-one	C_4_H_6_N_2_O	98
38.	9.535	0.09	Cyclopent-4-ene-1,3-dione	C_5_H_4_O_2_	96
39.	9.595	0.01	1-(4-Methoxy-2-nitroanilino)-1- a-d arabinofuranose	C_12_H_16_N_2_O_7_	300
40.	9.685	0.01	Butanoic acid, heptafluoro-, 4-butoxy-4-oxobutyl ester	C_12_H_15_F_7_O_4_	356
41.	9.998	0.31	2(5H)-Furanone	C_4_H_4_O_2_	84
42.	10.297	0.12	6-Oxa-bicyclo[3.1.0]hexan-3-one	C_5_H_6_O_2_	98
43.	11.215	0.01	11-Bromo-1-undecanol, TMS derivative	C_14_H_31_BrOSi	322
44.	11.479	0.01	2,4-Dihydroxy-2,5-dimethyl-3(2H)-furan-3-one	C_6_H_8_O_4_	144
45.	11.295	0.01	Decane, 3,4-dimethyl-	C_12_H_26_	170
46.	12.265	0.01	Cyclohexanone, 2-ethyl-4-methoxy-	C_9_H_16_O_2_	156
47.	12.360	0.02	Cyclotetrasiloxane, octamethyl-	C_8_H_24_O_4_Si_4_	296
48.	13.845	0.02	2,4-Di-tert-butylthiophenol	C_14_H_22_S	222
49.	14.075	0.05	Silane, 2-butenylmethoxymethylph	C_12_H_18_OSi	206
50.	14.155	0.07	Arsenous acid, tris(trimethylsilyl) ester	C_9_H_27_AsO_3_Si_3_	342
51.	14.250	0.02	2,2′-(Methylenedithio)bispropanoic acid	C_7_H_12_O_4_S_2_	224
52.	14.310	0.01	Benzene, 4-ethyl-1,2-dimethoxy-	C_10_H_14_O_2_	166
53.	14.840	0.01	1H-Pyrrole-2-ethanamine, 1-methyl-	C_7_H_12_N_2_	124
54.	14.965	0.02	1,2-Bis(dimethylphosphino)ethane	C_6_H_16_P_2_	150
55.	16.175	0.01	Zidovudine	C_10_H_13_N_5_O_4_	276
56.	17.715	0.02	l-Gala-l-ido-octose	C_8_H_16_O_8_	240
57.	20.125	0.01	DL-phenylalanine	C_9_H_11_NO_2_	165
58.	21.935	0.01	Fumaric acid, 2-chlorophenyl decyl ester	C_20_H_27_ClO_4_	366
59.	22.555	0.01	2-Methoxy-4-vinylphenol	C_9_H_10_O_2_	150
60.	24.350	0.01	Methyl abietate isomer	C_21_H_32_O_2_	316
61.	24.440	0.01	Isovanillic acid, 2TMS derivative	C_14_H_24_O_4_Si_2_	312
62.	24.762	0.20	Methyl 4-methoxysalicylate, TMS derivative	C_12_H_18_O_4_Si	254
63.	24.795	0.10	Resorcinol, 2TMS derivative	C_12_H_22_O_2_Si_2_	254
64.	25.145	0.02	3,5-Dinitrobenzyl alcohol, benzyldimethylsilyl ether	C_16_H_18_N_2_O_5_Si	346
65.	25.695	0.01	Cyclotetrasiloxane, 2,4,6,8-tetrame	C_4_H_16_O_4_Si_4_	240
66.	25.795	0.01	2-Furanone, 3,4-dihydroxytetrahydro	C_4_H_6_O_4_	118
67.	28.296	0.05	1,3-Benzenedimethanethiol	C_14_H_26_S_2_Si_2_	314
68.	29.740	0.01	Epimethendiol-diOTMS	C_26_H_48_O_2_Si_2_	448
69.	31.995	0.02	Ethyl-1-thio-.beta.-d-glucopyranosi	C_8_H_16_O_5_S	224
70.	32.500	0.06	6-Desoxy-l-gulitol	C_6_H_14_O_5_	166
71.	38.351	0.01	Ethyl iso-allocholate	C_26_H_44_O_5_	436
72.	39.620	0.01	d-mannitol, 1-decylsulfonyl-	C_16_H_34_O_7_S	370
73.	40.154	0.12	Hexadecanoic acid, methyl ester	C_17_H_34_O_2_	270
74.	44.621	0.03	1,1′-Bicyclopentyl, 2-hexadecyl-	C_26_H_50_	362
75.	47.395	0.02	Bicyclo[2.2.1]heptane, 2,2-dimethyl-5-methylene-	C_10_H_16_	136
76.	47.787	0.01	Oleic acid	C_18_H_34_O_2_	282
77.	48.545	0.01	Thiazolidine, 2-methyl-2-(4-nitrophenyl)-	C_10_H_12_N_2_O_2_S	224
78.	48.726	0.04	Methyl stearate	C_19_H_38_O_2_	298
79.	48.888	0.01	9,12-Octadecadienoic acid (Z,Z)-, methyl ester	C_19_H_34_O_2_	294
80.	49.202	0.18	cis-Vaccenic acid	C_18_H_34_O_2_	282

## Data Availability

Major part of the data is already included in the manuscript; the remaining data will be made available on reasonable request.
